# A practical exact maximum compatibility algorithm for reconstruction of recent evolutionary history

**DOI:** 10.1186/s12859-017-1520-4

**Published:** 2017-02-23

**Authors:** Joshua L. Cherry

**Affiliations:** National Center for Biotechnology Information, National Library of Medicine, National Institutes of Health, Bethesda, MD 20894 USA

**Keywords:** Phylogeny, Maximum compatibility, Homoplasy, Bacterial genomes

## Abstract

**Background:**

Maximum compatibility is a method of phylogenetic reconstruction that is seldom applied to molecular sequences. It may be ideal for certain applications, such as reconstructing phylogenies of closely-related bacteria on the basis of whole-genome sequencing.

**Results:**

Here I present an algorithm that rapidly computes phylogenies according to a compatibility criterion. Although based on solutions to the maximum clique problem, this algorithm deals properly with ambiguities in the data. The algorithm is applied to bacterial data sets containing up to nearly 2000 genomes with several thousand variable nucleotide sites. Run times are several seconds or less. Computational experiments show that maximum compatibility is less sensitive than maximum parsimony to the inclusion of nucleotide data that, though derived from actual sequence reads, has been identified as likely to be misleading.

**Conclusions:**

Maximum compatibility is a useful tool for certain phylogenetic problems, such as inferring the relationships among closely-related bacteria from whole-genome sequence data. The algorithm presented here rapidly solves fairly large problems of this type, and provides robustness against misleading characters than can pollute large-scale sequencing data.

**Electronic supplementary material:**

The online version of this article (doi:10.1186/s12859-017-1520-4) contains supplementary material, which is available to authorized users.

## Background

Reconstruction of phylogenetic trees from molecular sequence data has numerous applications. Diverse methods of reconstruction, which are adapted to different circumstances or make different trade-offs between speed and accuracy, are in use. Most of the commonly-used methods fall into three categories: distance methods, maximum likelihood, and maximum parsimony.

Less well known are methods based on maximum compatibility. Although this criterion was first described long ago [1, 2], compatibility methods have not seen much use, perhaps because their conditions of applicability have rarely been met. However, a compatibility criterion is attractive for certain applications made possible by high-throughput sequencing, in which extensive sequencing is performed on possibly large numbers of closely-related organisms. An example is whole-genome sequencing of very closely-related bacteria. Phylogenetic reconstruction from such data is important for, among other things, surveillance of bacterial pathogens.

Compatibility can most easily be compared to parsimony. Maximum parsimony methods seek a tree that minimizes the total number of changes of character state that are necessary to explain the data. Conventional maximum compatibility seeks a tree that minimizes the number of characters (e.g., alignment positions) required to have more than the minimum possible number of changes of state. These criteria, though related, are not equivalent, and can give different results. Suppose, for example, that the polymorphic subset of our data consists of 100 two-state characters. One tree topology requires two changes of state for each of 20 characters and just one change for each of the remaining 80. A second topology requires 50 changes of state for one character and just one change for each of the remaining 99 characters. Maximum parsimony prefers the first topology (120 state changes rather than 149), whereas maximum compatibility prefers the second (99 compatible characters rather than 80). Which preference is scientifically justifiable?

The answer depends on the context. In the case of bacterial pathogen surveillance, incompatible characters are expected, and generally observed, to constitute only a small fraction of the variable sites (see, e.g., [3] and Results). The 100 variable sites in the example would typically be accompanied by several million sites in the genome that were not observed to vary at all, suggesting that few if any sites will have changed more than once by ordinary sequence divergence. On the other hand, there are many ways in which misleading or incorrect character states can come to pollute the data, and the affected characters often require multiple changes of state on any reasonable tree. Sources of problematic characters include genetic recombination, misassembly or misalignment due to unrecognized repeat sequences, and contamination of samples by material from related bacteria. Thus, the topology preferred by maximum compatibility corresponds to what may be the simplest explanation of the data: a single corrupted alignment column, which conveys no useful information, along with 99 variable sites with a single change of state.

Felsenstein [4] gave conditions for applicability of maximum compatibility. Scotland and Steel [5] exhibited conditions under which compatibility would be superior to parsimony, and suggested application of compatibility to morphological traits. An application to sequence data is described by Gupta and Sneath [6].

In the simplest case for maximum compatibility, all characters are binary (e.g., no more than two different nucleotides occur in any alignment column) and there are no ambiguous character states. Under these conditions, pairwise compatibility among a set of characters guarantees compatibility of the set as a whole with some tree [7]. The maximum compatibility problem then becomes a maximum clique problem. Although the maximum clique problem is NP-hard [8], existing algorithms can solve instances of moderate size in reasonable time (reviewed in [9]), and various speed enhancements apply to the type of instance involved in this phylogenetic application (e.g., the graph is typically very densely connected because most pairs of characters are compatible).

In practice, neither of these conditions holds. Characters are not always binary; a sequence column may contain three or all four bases. Ambiguous states may occur. Indeed these are abundant in the bacterial genome use case (see, e.g., Results).

Failure of the first condition is of little consequence. Under the conditions that make compatibility appealing, only a small fraction of the variable sites are expected to contain more than two bases, and those that do are highly suspect. Non-binary sites are indeed very rare in the motivating case of closely-related bacteria (see Results). These sites are simply discarded by the algorithm presented here. The algorithm thus applies a modified compatibility principle: maximize the number of characters that do not require more than one change of state. Characters with more than two observed states can never satisfy this criterion. This is the form of maximum compatibility assumed by Felsenstein [4] when assessing it from the perspective of maximum likelihood.

Ambiguous character states, on the other hand, cannot be ignored or discarded. The algorithm presented here nonetheless provides an exact solution to the modified compatibility problem in the presence of ambiguities. It solves a maximum clique problem and, if the result is not a solution to the phylogenetic problem at hand, iteratively modifies the problem until such a solution is reached. It then resolves ambiguities in the compatible character set to the extent possible, and produces a corresponding phylogenetic tree with branch lengths.

## Methods

### Maximum compatibility algorithm

The algorithm described here solves a version of the maximum compatibility problem. A character is said to be compatible with a tree if, for some resolution of any ambiguous states for that character, the character states can be explained with at most one change of state on that tree. A set of characters is said to be compatible if there exists a tree with which all of them are compatible. A maximum compatible set of characters is a compatible set that is at least as large as any other compatible set. The algorithm takes a nucleotide character matrix as input and finds all maximum compatible character sets. It then produces a strict consensus tree that reflects all maximum compatible character sets.

**Fig. 1:**
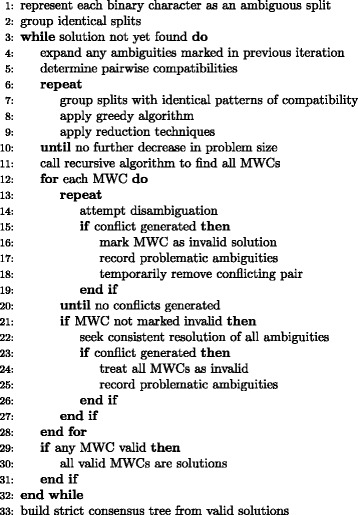
Outline of the overall algorithm

**Fig. 2:**
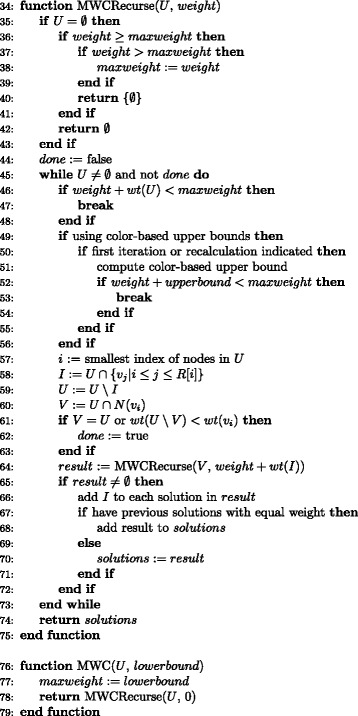
The recursive maximum weight clique algorithm. A\B indicates the set difference between A and B, i.e., the set of all elements of A that are not elements of B. *N*(*v*) indicates the set of all vertices that are neighbors of (i.e., compatible with) vertex *v. wt(x)* indicates the weight of a vertex or the sum of the weights of a set of vertices. *R*[*i*] indicates the index of the end of the range of vertices following *v*_*i*_ that share its pattern of conflicts with all vertices *v*_*j*_ with *j* > *i* (see text)

The algorithm (Figs. 1 and 2) is based on a variant of established algorithms for the maximum weight clique (MWC) problem. Vertices of the usual maximum clique problem are combined into a single weighted vertex whenever they have identical patterns of compatibility among the other vertices. Several modifications greatly increase the speed of this core computation. The larger algorithm handles various complications of ambiguous character states. It produces a phylogenetic tree and reports statistics on the dispositions of characters. Optional output includes a list of the characters that failed to meet the compatibility condition.

#### Data representation

Initial input consists of a nucleotide character matrix. This may be a complete multiple sequence alignment, but often only a discontiguous set of sequence positions is given, and their order is of no importance. Each column of the matrix corresponds to a character (a sequence position), and each row corresponds to a terminal node on the tree (e.g., a sequenced genome). Allowed character states are the four DNA bases (A, G, C, and T) and the ambiguity symbol N.

The columns of interest are those in which exactly two of {A, G, C, T} occur, possibly along with occurrences of N. Columns with three or four unambiguous bases would require more than one change of state regardless of tree topology, and therefore are excluded. Columns that contain only one unambiguous base do not require any changes of state, and are also discarded.

Each column of interest specifies a split (a binary partition) of the terminal nodes that may be ambiguous. A column containing {A, G} (or any other unambiguous pair) splits the terminal nodes unambiguously into two sets in the obvious way. For a column with {A, G, N}, set membership is ambiguous for the rows containing N.

**Fig. 3:**
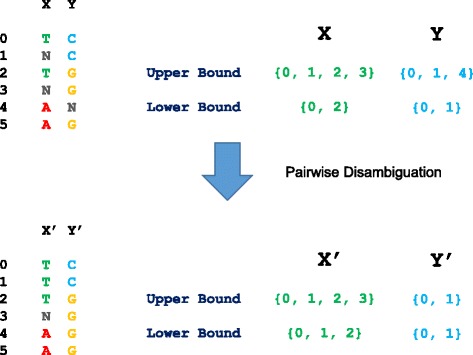
Illustration of treatment of ambiguous character states. Two nucleotide characters having ambiguities (*top left*) are represented by pairs of sets (*top right*). The column designated “X” is represented by *upper* and *lower* bounds on the set of rows with “T”, although it could instead be represented by the set with “A”. Similarly, “Y” is represented in terms of the rows with “C”. The result of disambiguation using only information from this pair is shown at the *bottom*, including the implied effect on the nucleotide character states

A (possibly ambiguous) split is represented by lower and upper bounds on one of its two component sets, as illustrated in Fig. 3. Each bounding set is represented by a sorted array of unsigned integers, each integer being the index of a terminal node (equivalently, a row of the character matrix). The choice of which set to take as the representative–e.g., the set of leaves with A at a particular position or the complementary set with G–may be made arbitrarily, and the algorithm may take the complement of a set when it is convenient to do so. The operation of complementation of an ambiguous set consists of interchanging the upper and lower bounds and complementing each of them.

Columns corresponding to the same ambiguous split are combined and represented as a split with an associated column count. The resulting collection of ambiguous splits with counts contains all of the information used in subsequent steps.

#### Pairwise compatibilities

Pairwise compatibilities are determined for all pairs of splits present in the data. Two splits represented by *X* and *Y* are compatible if, and only if, at least one of four conditions holds:

1. The upper bound on *X* is a superset of the lower bound on *Y*.
2. The lower bound on *X* is a subset of the upper bound on *Y*.
3. The lower bound on *X* and the lower bound on *Y* are mutually exclusive (their intersection is the null set).
4. The upper bound on *X* and the upper bound on *Y* are jointly exhaustive (their union is the universal set, i.e., the set of all terminal nodes).

In Fig. 3, for example, condition 1 is met, but none of the others holds.

Note that if any of these conditions is met, any other is met under complementation of *X*, complementation of *Y*, or complementation of both. They represent a single underlying condition that can manifest in four ways, depending on two arbitrary choices between complementary sets as representatives of the two splits. Condition 1 is obviously a sufficient condition for some set within the bounds of *X* to be a superset of some set within the bounds of *Y*. It is also a necessary condition: if the superset relation holds between any such sets, it must hold between the largest possible value for *X* and the smallest for *Y*. The disjunction of the four conditions therefore corresponds to the possibility of resolving ambiguities such that the resulting unambiguous splits are compatible, i.e., at least one member of each split is a subset of one member of the other split.

The compatibility condition given above means that some resolution of the ambiguities in *X* is compatible with some resolution of the ambiguities in *Y*. It does not imply that all resolutions of *X* are compatible with all, or even some, resolutions of *Y*. Thus, a set of splits that are pairwise compatible in this sense are not necessarily mutually compatible: there may be no resolution of all ambiguities that preserves all pairwise compatibilities, and (equivalently) no tree topology that is compatible with the entire set. On the other hand, the compatibility constraints put on one split by another allow resolution of much ambiguity once a mutually compatible set has been found.

#### Preprocessing

Although the matrix of pairwise compatibilities described above could be used directly as the connectivity matrix for a maximum weight clique problem, several intervening steps allow for greatly improved speed.

Distinct splits may exhibit identical patterns of compatibility with the other splits present in the data (equivalently, different rows of the compatibility matrix may be identical to one another). These are combined for the purpose of finding a maximum weight clique, which necessarily contains either all or none of them. This procedure can greatly reduce the size of the problem to be solved.

Next, a greedy algorithm is run to obtain an approximate solution to the problem. In each iteration, a vertex with the largest total weight of conflicts with the remaining vertices is removed from the set until no conflicts remain. This procedure serves several purposes. First, the resulting clique provides a lower bound on the weight of the optimal solution. This bound allows the exact algorithm to run more quickly by rapidly rejecting families of solutions that could not possibly be as good as the approximate solution. The bound also allows up-front removal of vertices that conflict with too many columns to be part of an optimal solution, which may in turn allow for further consolidation of vertices with identical conflict patterns. It also allows identification of vertices that must be included in any optimal solution; these are marked for inclusion and removed from the problem. Second, the greedy algorithm provides an ordering of the vertices that can greatly increase the speed of the exact algorithm: vertices are presented in order of removal, followed by the vertices that form the remaining clique. This ordering is broadly similar to that employed by [10] in that vertices with lower connectivity (more conflicts) tend to come earlier.

#### Problem size reduction

The size of the problem to be solved is further reduced before an exact solution is sought. Vertex coloring provides, for every vertex, an upper bound on the weight of a clique that contains it [11]. Together with the lower bound on the MWC, this information allows elimination of many vertices from consideration before the main computation is performed. A more drastic reduction is achieved by consideration of weights. If the weight of a vertex exceeds the combined weight of the vertices that conflict with it, the first vertex must be included in any maximum weight clique and the conflicting vertices must be absent from it. This is so because any clique not containing the first vertex would be made larger in weight by addition of the first vertex and removal of any vertices that conflict with it. Thus, several vertices can be removed from the problem; one is recorded as necessarily included, and the others are discarded. This procedure is repeated until no additional vertices can be removed.

If the problem remains sufficiently large, a further reduction is applied, based on an extension of the reasoning given above. Any subset of a clique, including the set of members that conflict with a particular vertex, must itself be a clique. Thus, a vertex is recognized as necessarily included in any MWC, and the vertices that conflict with it are removed from consideration, if the first vertex outweighs all cliques that can be formed from the conflicting vertices. This determination is made using a modified version of the exact MWC algorithm that returns as soon as it finds a clique of sufficient weight. This can be much faster than finding the MWC, whose weight may far exceed the required minimum.

Size reduction may enable further grouping of columns on the basis of shared patterns of conflict. This additional grouping may in turn allow additional size reduction. Thus, the combination of preprocessing (which includes grouping) and size reduction is performed iteratively until no further reduction occurs (lines 6–10).

#### Exact maximum weight clique algorithm

Maximum weight cliques are found by a version of the algorithm of Carraghan and Pardalos [10] (see [12], Algorithm 1, for pseudocode), adapted to the weighted variant of the maximum clique problem (Fig. 2). When there is more than one maximum weight clique, this version returns all of them. Branch cuts may be performed based on upper bounds on clique weight calculated in either of two ways described below. Several modifications increase the speed of the algorithm:

- As noted above, a greedy algorithm provides a vertex ordering that speeds computation.
- The lower bound on maximum clique weight, provided by the greedy solution, greatly speeds the computation.
- An additional cut condition is applied to the iterative removal of vertices (lines 61–63): when the weight of the first vertex in a candidate list exceeds the total weight of its conflicting vertices within that list, there is no need to continue beyond the current iteration. This is so because subsequent iterations consider only cliques that do not contain the first vertex, but any MWC contained in the candidate list must include the first vertex under these conditions (because otherwise addition of the first vertex and removal of conflicting vertices must yield a clique of larger weight).
- A vertex may be immediately followed in the ordering by one or more vertices that share its pattern of connectivity to all other vertices that come after it. At the point in the algorithm where the first remaining vertex is removed from the candidate set and provisionally included in a clique, any such following vertices that remain in the candidate set are removed and considered for inclusion along with it (lines 58–59). This is justified because any MWC of the candidate set that contains the first vertex must contain these additional vertices as well, since it would otherwise be possible to add them without introducing conflicts, yielding a clique with larger weight.

Branch cuts may also be based on vertex coloring, essentially as described by Tomita and Yamada [11]. If the upper bound on clique weight calculated for a candidate set is too low to allow the current best solution to be equaled, the branch is terminated (lines 50–55). Otherwise, the margin by which the upper bound exceeds the requirement is recorded. Because it is costly, an upper bound calculation is not performed again until the decrease in candidate weight, combined with any increase in the maximum observed clique weight, exceeds this margin.

When this option is in force, a coloring is calculated for each terminal sequence of vertices before the main algorithm is run. The algorithm then uses the appropriate coloring according to the first vertex in the subsequence that it is evaluating. Compared to the use of a single coloring throughout, this approach yields generally tighter bounds and hence greater speed.

An alternative approach can provide tighter upper bounds and hence increased speed. The usual approach using vertex coloring considers only conflicts among nodes in the same color class. The alternative described here incorporates some of the remaining conflicts, which involve vertices in different color classes. The color classes are arranged into a tree, constructed so that pairs of classes containing vertices with “important” conflicts—those involving the vertices with highest weight in their color classes and overall—tend to be adjacent in the tree (see below). For a given set of vertices, the algorithm calculates the maximum weight of any subset that respects all conflicts between vertices in tree-adjacent classes in addition to within-class conflicts. This is accomplished through dynamic programming. For any color class, *n* + 1 scores are calculated, where *n* is the number of vertices in that class. These scores correspond to the *n* + 1 choices of which vertex, if any, to include from that class. Each score represents the maximum weight, given that choice, of a subset that satisfies the constraints but includes only nodes from the subtree defined by that color class. The score is set to zero for any vertex not present in the set under evaluation. Scores for all classes are calculated recursively, and the maximum score for the root of the tree is the desired overall maximum. Each such evaluation is costlier than the usual coloring-based calculation, but it can yield much tighter upper bounds, and hence earlier branch cuts and dramatic improvements in overall speed.

A color class tree is constructed as follows for each set of color classes (and hence for each terminal sequence of vertices) before the main algorithm is run. The color classes are first ordered by decreasing maximum member weight. They are then added to the tree by alternation of these steps:

1. The first remaining (not yet added) color class is added to the tree. If it is the first class to be added, it becomes the root. Otherwise, it is attached as a child of the root.
2. Any remaining classes whose highest-weight member conflicts with the highest-weight member of the class added in (1) are added as children of that class. The added children are then treated, in order, in the same way, so that they may acquire children of their own and more distant descendants.

A preference for enforcement of “important” conflicts, as defined above, is accomplished by step 2 in conjunction with the class ordering.

By default, the algorithm chooses whether to calculate upper bounds, and, if so, by which method, on the basis of the size of the problem. The method used can also be specified by the user.

#### Disambiguation

The above procedure yields a collection of splits that generally contain ambiguities. Ambiguities in a split may often be resolved by constraints imposed by other splits in the collection. Ambiguities are resolved to the extent possible by an iterative pairwise process, and only those splits that are fully resolved by this process impose splits on the computed phylogenetic tree or contribute to the lengths of its branches.

Consider a pair of possibly ambiguous splits, represented by set ranges *X* and *Y. X* may resolve some ambiguities in *Y*, and *vice versa*, when exactly one of the four compatibility conditions (see above) holds. Suppose, for example, that only condition (1) holds. This implies that, on any consistent resolution of ambiguities, *X* is a superset of *Y*. It follows that *Y* can be no larger than the upper bound on *X*. We may, therefore, obtain a stricter upper bound on *Y*, namely the intersection of the original upper bound on *Y* and the upper bound on *X*. Similarly, the lower bound on *X* is replaced by its union with the lower bound on *Y*. Figure 3 illustrates the application of this rule and the corresponding implicit effects on ambiguous nucleotide states. Analogous disambiguation rules are applied for the other three compatibility conditions. Like the compatibility conditions themselves, these four rules are in reality a single underlying rule applied to different representations of the data. These operations correspond to Meacham’s [13] second rule for partial splits.

The pairwise disambiguation procedure is performed iteratively on pairs of splits until no further disambiguation is possible. If this condition is reached without the introduction of any pairwise incompatibilities, the disambiguated splits are converted to a phylogenetic tree with branch lengths (or contribute to a consensus tree). If a pairwise inconsistency does arise, a modified MWC problem is solved, as described next.

When ambiguities are not completely resolved, there may be total splits implied by the data that are not recovered by pairwise disambiguation. For the data on which the algorithm has been tested, the vast majority of ambiguous splits are fully resolved (see Results), so there are few, if any, of these. Nonetheless, alternative procedures may be worth pursuing.

#### Handling false solutions

As noted above, in the presence of ambiguities a maximum weight clique need not be a solution to the maximum compatibility problem: there may be no way to resolve all ambiguities such that pairwise compatibility remains intact. The disambiguation procedure may therefore give rise to incompatibilities. When this occurs for all of the cliques found, it is necessary to find a different candidate solution to the compatibility problem. This is done by solving a modified instance of the MWC problem.

To understand how this situation is handled by the algorithm, it is helpful to consider a correct but inefficient means by which ambiguities could have been handled. In this impractical approach, each ambiguous split containing *n* ambiguities is expanded into all 2^*n*^ unambiguous possibilities that it represents. These are marked as incompatible with one another, so that at most one resolution of any ambiguous split is included in any clique. Other compatibilities are determined as above. Solution of the resulting maximum weight clique problem yields a solution to the maximum compatibility problem.

Such a procedure would be practical only if ambiguities were rare and reasonably evenly distributed across matrix columns. When some columns contain many ambiguities, the number of vertices needed to represent all possibilities becomes prohibitively large. However, limited expansion of ambiguities, guided by the incompatibilities that arose in the course of disambiguation, allows reasonably rapid calculation of another candidate solution, as described below.

Suppose that disambiguation produces pairwise incompatibilities. This occurrence identifies at least one pair of splits that were originally compatible (since they were in the maximum weight clique) but became incompatible in the course of disambiguation. Expansion of just these two splits into their component possibilities would guarantee a different outcome: if solution of the modified maximum weight clique problem results in any incompatibilities, these will involve different splits, which can then also be expanded. Furthermore, the acquired incompatibility can be attributed to subsets of ambiguities in the original pair whose resolution destroyed one or more compatibility conditions. Suppose, for example, that the two original ambiguous sets *X* and *Y* satisfied only compatibility condition 1, and that the disambiguation process yields incompatible restrictions *X’* and *Y’*. Then the implicated ambiguous elements for *X* are those absent from the upper bound of *X’* but present in the lower bound of *Y’*. These are precisely those elements whose exclusion from *X’* make it too small to be a superset of any resolution of *Y’*, so that condition 1 is not satisfied.

Thus, complete expansion of an implicated split into 2^*n*^ unambiguous possibilities is not necessary. In fact, expansion into just two possibilities that resolve only one ambiguous element may prevent the conflict and avert a costly combinatorial explosion. Therefore, for each split in a conflicting pair the algorithm chooses one element for expansion (namely, the smallest implicated index). If there are multiple MWCs, however, they may implicate different elements of the same split. In general, then, a split is expanded into 2^*m*^ possibilities, with *m* < = *n*. If *m* < *n*, these possibilities are themselves ambiguous (each contains *n–m* ambiguities). Incompatibilities in solutions of the modified MWC instance will necessary involve other splits or different ambiguities in these splits.

The above procedure designates one or two splits for expansion at certain ambiguities. Disambiguation is then attempted on what remains of the clique after these splits are removed, and the procedure is repeated until no incompatibilities remain (lines 13–20). This is not necessary for correctness, but may identify additional splits for expansion without an additional MWC search and hence improve performance. The result is a set of one or more ambiguous splits that are to be partially expanded with respect to certain designated ambiguities.

If solution of the modified problem also yields incompatibilities upon disambiguation, splits/ambiguities are again designated for expansion and the process is repeated. Among the splits to be expanded may be the products of previous expansions. Iteration (lines 3–32) must eventually yield a legitimate solution to the maximum compatibility problem. In practice this requires at most a few iterations and only modest enlargement of the problem, and computations complete in reasonable times.

When pairwise disambiguation succeeds without conflict, the splits may nonetheless lack mutual compatibility. This situation has not been encountered with real data during the development of the algorithm. Nonetheless, the algorithm checks every candidate solution by seeking a complete and consistent resolution of all ambiguities, the existence of which ensures mutual compatibility. First, any of the remaining ambiguous splits that can be resolved to singletons (splits corresponding to terminal branches) are so resolved, eliminating the possibility of conflicts involving them. Second, any ambiguous splits that can be resolved to unambiguous splits already in the set are resolved in that way. This procedure cannot introduce new conflicts, so it preserves mutual compatibility. Finally, remaining incompatibilities are resolved by iteratively resolving one ambiguous element arbitrarily and performing pairwise disambiguation on the modified set. Iteration proceeds until there are no ambiguities remaining or a conflict arises.

A conflict at this stage is treated much like a conflict arising in the earlier disambiguation of the original set: one or both of the splits involved are marked for partial expansion in a subsequent MWC search (line 25). However, when there are multiple maximum cliques, a conflict at this stage for *any* of those cliques mandates a subsequent search, even if some other solutions proceed without conflict (line 26). This is because the search for a compatible resolution of all ambiguities is not guaranteed to succeed even if one exists. It has not been observed to fail, except on artificial data constructed to make it do so, but the possibility is handled appropriately.

If no conflict arises, the result is a complete resolution of all ambiguities that is pairwise compatible and hence mutually compatible. This resolved set serves as a proof of the mutual compatibility of the original set. A tree corresponding to the fully resolved set may optionally be produced as auxiliary output. However, it is not used for the main tree, which is based on pairwise disambiguation results.

#### Multiple maximum cliques

An instance of the maximum clique problem may admit multiple solutions. The exact search described here is exhaustive and may yield more than one clique of the same size. All of the solutions are evaluated for mutual compatibility as described above. Any incompatibilities that arise from different solutions are combined appropriately for determining any subsequent ambiguity expansions. If one or more solutions are found to possess mutual compatibility, these represent the largest sets of mutually compatible columns, provided that all of the other solutions produced incompatibilities during pairwise disambiguation. The pairwise-disambiguated maximum compatible sets are then used for tree construction.

Although a tree can be derived from each such result, by default they are combined to produce a consensus tree. The consensus tree contains exactly those splits that are found in all of the solutions. The length of the branch corresponding to a split is the minimum of the split’s total count among the solutions.

#### Implementation

The algorithm was implemented in C++ and Python, with an interface between the two generated by SWIG [14]. It was developed under Linux. Source code and build instructions are available at [15].

### Bacterial sequence data

The algorithm was assessed using bacterial nucleotide character matrices derived from whole-genome sequence data. These character matrices were produced by the NCBI pathogen detection pipeline. Information about this pipeline can be found at [16] and [17]. The character matrices analyzed are available at [15].

### Maximum parsimony trees

Maximum parsimony trees were built with tnt (Willi Hennig Society edition) [18]. One hundred replications were performed with the xmult command, with five runs each of the parsimony ratchet. A consensus tree was then built after trees were collapsed with TBR.

## Results

### Application of maximum compatibility to bacterial genomes

The algorithm described here was applied to *Salmonella enterica* data generated by the NCBI pathogen detection pipeline. Data consisted of nucleotide character states for sets of *Salmonella* genomes (hundreds or thousands of variable sites for up to 1854 genomes). Each set consists of closely-related genomes (largest tree distances between isolates are approximately 150–250 nucleotide differences), and is therefore referred to as a cluster. Character states were derived from raw sequence reads where available, but some genome assemblies were also included. Character states had been filtered to remove several kinds of potentially problematic sites: those with large numbers of conflicting sequence reads, those with low coverage, certain repeat sequences, and sites in regions with high densities of sequence differences (suspected to represent recombination events, inaccurate sequence, or other problems). Table 1 gives the run times for the twenty largest clusters, along with information about the input data and the disposition of columns in the tree reconstruction process.

**Table 1:** Summary of results and performance on *Salmonella* data

Cluster number	Number of Genomes	Columns in Input			Maximum Compatible Set(s)		Ambiguities in Binary Columns		Columns Not Fully Disambiguated		Columns Represented on Tree			Execution time (sec.)
		Variable	Binary	Informative	Size	Number	Fraction Ambiguous States	Fraction of Columns with Ambiguities	Total	Informative and Non-redundant	Number	Fraction of variable	Fraction of binary	
1	1854	12237	12013	4933	11551	32	0.4%	72.1%	140.5	14	11410.5	93.2%	95.0%	9.77
2	1586	14229	14184	5851	13923	2	0.2%	43.3%	153	19	13770	96.8%	97.1%	3.92
3	943	7130	7121	2357	7032	2	1.4%	23.7%	168.5	2	6863.5	96.3%	96.4%	1.4
4	843	2439	2434	1206	2404	2	0.3%	8.9%	11	0	2393	98.1%	98.3%	0.48
5	826	6143	6134	1964	6014	1	0.9%	53.6%	189	3	5825	94.8%	95.0%	1.34
6	818	8271	8257	2524	8182	1	0.5%	41.1%	190	36	7992	96.6%	96.8%	1.43
7	450	6031	6023	2158	5973	2	0.7%	42.6%	93	0	5880	97.5%	97.6%	0.74
8	403	6323	6317	2386	6278	1	0.7%	39.5%	76	0	6202	98.1%	98.2%	0.6
9	379	5171	5164	1714	5002	1	1.2%	56.4%	148	0	4854	93.9%	94.0%	0.78
10	278	1156	1156	405	1145	1	0.6%	10.6%	8	1	1137	98.4%	98.4%	0.26
11	259	4223	4221	1279	4201	2	0.4%	10.6%	24	0	4177	98.9%	99.0%	0.35
12	215	3538	3533	1202	3517	1	0.8%	16.4%	39	1	3478	98.3%	98.4%	0.33
13	210	2879	2867	687	2824	2	0.7%	11.1%	18	0	2806	97.5%	97.9%	0.29
14	195	2697	2695	1223	2684	1	1.8%	44.4%	52	0	2632	97.6%	97.7%	0.42
15	193	2086	2084	786	2065	2	1.1%	12.4%	39	0.5	2026	97.1%	97.2%	0.46
16	192	903	902	276	874	4	4.7%	23.5%	47.5	3	826.5	91.5%	91.6%	0.26
17	190	2640	2638	984	2603	1	1.4%	12.0%	51	2	2552	96.7%	96.7%	0.29
18	171	1035	1033	353	1023	1	0.5%	8.2%	6	0	1017	98.3%	98.5%	0.23
19	171	979	979	337	968	1	1.7%	21.2%	30	2	938	95.8%	95.8%	0.26
20	168	2738	2738	612	2708	1	1.3%	11.9%	44	1	2664	97.3%	97.3%	0.28

Some counts are averages over multiple maximum compatible sets, and may therefore be non-integral

Tree computation was rapid. For the largest cluster, which contained 1854 isolates and had 4933 informative characters, the algorithm took just under 10 s to run using a single core on commodity hardware (Intel Xeon E5-2650 @2.6GHz). Calculating all 20 trees took a total of less than 30 s.

In all cases the maximum compatible set included well over 90% of the variable characters. After the pairwise disambiguation process, the vast majority of these were completely unambiguous. These facts lend support to the applicability of maximum compatibility, and suggest that any loss of information due to genuine homoplasy or inadequate disambiguation is small.

In the majority of the columns that could not be completely disambiguated, one of the unambiguous bases was present in only one row, making those columns uninformative for tree topology. This is true of columns in general (much of the total tree length is in the terminal branches), but is disproportionately common among those whose ambiguities are not all resolvable. Of the remaining such columns, the majority admit resolution to the same split as some fully disambiguated column, so they cannot imply any split that is not known from another column. Thus, only a very small number of columns not fully disambiguated are both informative and non-redundant.

The algorithm was also applied to data for other bacteria produced by the NCBI pathogen detection pipeline. Additional file 1: Table S1 shows results for all clusters with 100 or more members, after removal of isolates with more than 10% ambiguities for some taxa. These clusters represent eight genera belonging to a variety of bacterial groups. The results are similar to those for *Salmonella enterica*. Execution times were at most 1.2 s, and the vast majority of variable sites are compatible with the inferred trees and contribute to branch lengths.

### Maximum parsimony trees

Trees for each *Salmonella* cluster were also calculated by maximum parsimony for the purpose of comparison. These were similar in topology to the maximum compatibility trees, but for some clusters differed significantly.

An example with significant differences is cluster 1. The compatibility tree for this cluster displays 2786 splits, and the parsimony tree displays 2822. Because 1854 of these correspond to terminal branches, the numbers of informative splits are 932 and 968. Of these, 74 found on the compatibility tree are absent from the parsimony tree, and 110 found on the parsimony tree are absent from the compatibility tree. If we restrict attention to splits on one tree that conflict with those on the other (as opposed to merely resolving a multifurcation on the other tree), these numbers are 69 for compatibility and 79 for parsimony. These differences represent a significant fraction of the informative splits.

Analysis revealed that several nucleotide sites are highly homoplastic with respect to both trees. These generally required fewer changes on the parsimony tree than the compatibility tree. For example, the most homoplastic site according to the compatibility tree requires 59 changes, but the same site requires only 28 changes on the parsimony tree.

It is not surprising that parsimony arrived a tree topology that decreases the number of changes required. However, the requirement for 28 changes even according to parsimony, where most variable sites require just one change, suggests that this site is unreliable and should be ignored. Compatibility effectively ignores it, as it counts the 59 changes as no worse than two, whereas parsimony accommodates it, counting the reduction from 59 to 28 as a major improvement that would justify the loss of compatibility with multiple sites.

Additional sites show a similar pattern. The next most homoplastic sites according to compatibility require 54, 49, 47, 41, and 38 changes. On the parsimony tree, these counts are also reduced drastically, to 24, 34, 26, 31, and 30, respectively.

Further investigation traced most of the highly homoplastic sites to the minority of bacterial isolates for which raw sequence reads were unavailable. For these isolates there was no opportunity to remove uncertain base calls on the basis of read alignments, and the analysis relied on whatever assembly method had been used by the sequence submitters. Removal of these isolates eliminated most of the highly homoplastic sites and brought the compatibility and parsimony topologies into near agreement. This is additional evidence that the highly homoplastic sites are unreliable and should be ignored, as effectively done under maximum compatibility, and therefore that the compatibility tree is preferable to the parsimony tree.

### Effects of addition of suspect character data

A second type of comparison between compatibility and parsimony considers the effect of allowing normally excluded nucleotide sites into the input data. In a series of computational experiments, randomly chosen positions that had been removed by one form of filtering were re-introduced into the analysis, and the effects on compatibility and parsimony trees were compared. Removal of these sites had been based on identification of sequence differences that cluster on the genome. Such clusters can result from various biological events and technical problems and are likely to be misleading.

The starting point for addition of these characters consisted of those sites that were compatible with both the original compatibility tree and the original parsimony tree. For this set of sites, the two methods yield the same tree topology, which is a consensus between the two original trees. This tree serves as a common basis of comparison for the effects of added sites on compatibility and parsimony. Any split that conflicts with this tree also conflicts with the original trees produced by both methods.

**Fig. 4:**
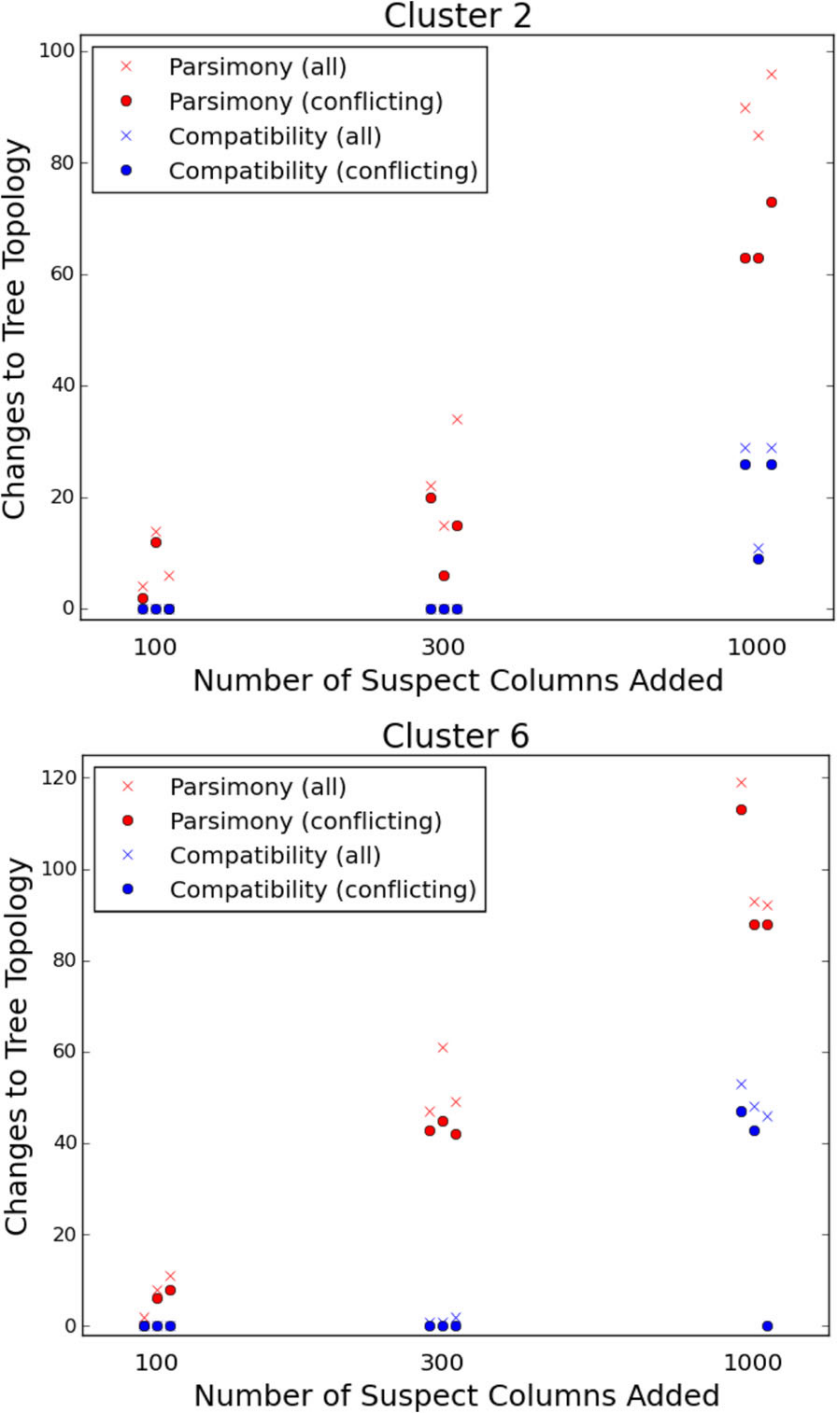
Susceptibility of maximum compatibility and maximum parsimony to the influence of suspect sequence data. The effect of adding 100, 300, or 1000 columns of suspect data is shown for each method. Three replicates (independent random selections of columns) are shown for each number of columns. The topological difference between trees reconstructed with and without the suspect columns is quantified by the number of columns on either tree that are absent from the other and the number on either tree that conflict with the other

Results are shown in Fig. 4 for clusters 2 and 6, the two clusters most subject to the effects of the suspect sites (as judged by their effect on compatibility trees, so as to avoid any bias against parsimony). As more randomly chosen suspect sites are added, the inferred tree topologies increasingly change. Parsimony is clearly more susceptible to the effects—presumably detrimental—of the added sites, whereas compatibility is much more robust to them.

### Algorithm comparisons

The Phylip phylogentic software package [19] includes a program, dnacomp, for maximum compatibility tree reconstruction from nucleotide data. Applied to the twenty *Salmonella* clusters (binary characters only) with the default parameters, dnacomp took much longer to run than the algorithm described here. Run times ranged from several seconds for the smallest clusters (compared to several tenths of a second) to nearly ten hours for the largest (compared to approximately ten seconds). In several cases, dnacomp results fell slightly short of the true compatibility maximum.

The techniques described here for reducing problem size (see Methods) often reduce the phylogenetic problem to a trivially small (or even zero-size) instance of the maximum weight clique problem. In cases where a non-trivial instance remained, the recursive MWC algorithm described here, with either use of vertex coloring, was found to be much faster than the algorithm of Östergård [20, 21] for solving the reduced problem. This performance advantage is presumably dependent on characteristics of the problem instances that occur in this application. It is noteworthy, however, that the reduced instances are not necessarily very densely connected.

## Discussion

The algorithm described here computes an exact maximum compatibility consensus tree for binary characters in the presence of ambiguity. The computation is rapid for character data with the intended properties. For input derived from whole-genome sequencing of closely-related bacteria, consisting of up to several thousand informative sites for nearly 2000 genomes, trees were computed in a few seconds or less. Compatibility is thus a computationally feasible phylogenetic method for such data.

The speed of the algorithm will vary with attributes of the input data besides its size. As expected for an NP-hard problem, worst-case run time can be long for large problem sizes. The high speed of the bacterial tree computations is made possible by the nature of the biological input data. Most important would seem to be the high density of the compatibility graph, i.e., the fact that vast majority of character pairs are compatible.

The main motivation for applying compatibility in this context is to make phylogenetic inference more robust to problematic sequence positions. Computational experiments confirmed that compatibility is more resistant than parsimony to the effects of misleading sequence positions on tree topology. These experiments were based on the effects of actual sequence data that is normally identified as suspicious and discarded.

Maximum compatibility may be compared to another approach to misleading sequence positions. Some analyses of bacterial genomes have discarded any positions that are incompatible with an initial tree calculated by a more commonly used phylogenetic method [3, 22]. This procedure may be helpful, but it is not a substitute for the benefits of maximum compatibility. If the initial tree topology is incorrect due to problematic sites, the set of positions chosen for retention by this procedure will also be incorrect. Re-computation of the tree based on this misidentified subset does not remedy the situation. It will, in fact, tend to reproduce the original flawed tree topology or a less-resolved version of it (this outcome is guaranteed, for example, under maximum parsimony). With maximum compatibility, in contrast, identification of potentially misleading sites is an integral part of determination of the tree topology, not a subsequent step that is determined by a topology that may be corrupted by those very sites.

Compatibility methods and whole-genome sequencing appear to be particularly well matched. Compatibility is not frequently used for analysis of nucleotide sequences, perhaps because it is not often appropriate. It is most applicable when most of the variable sites have changed just once over the length of the true tree. In practice this means that the vast majority of sequence positions do not vary in the group under study, so that the number of useful characters will be small unless many nucleotide positions are sequenced. Whole-genome sequencing provides data for millions of positions, allowing for differentiation of closely related isolates that are identical at all but a tiny fraction—but a reasonably large number—of nucleotide sites. It is, however, subject to many sources of misleading character states, including recombination events, unrecognized repeat sequences, systematic sequencing errors, and contamination of sequence reads, that can overwhelm the truly informative sites. Maximum compatibility provides robustness against these phenomena.

## Conclusions

The maximum compatibility algorithm presented here rapidly computes phylogenies of closely-related bacteria from genome sequence data. This application domain appears to conform to the assumptions of maximum compatibility: the vast majority of variable characters require only one change of state on the phylogenetic tree. In the presence of moderate levels of sequence ambiguity, the method is able to resolve most ambiguous states. Compared to maximum parsimony, the method is robust to phylogenetically misleading nucleotide positions that can be found in actual data. It may therefore be a preferred method for an important class of phylogenetic problems.

## Additional file

Additional file 1: Table S1. Summary of results and performance on bacteria other than *Salmonella*. (PDF 311 kb)

## Abbreviations

MWC: Maximum weight clique
