# Supplementary material for: A practical exact maximum compatibility algorithm for reconstruction of recent evolutionary history
Source: BMC Bioinformatics. 2017 Feb 23;18:127. doi: 10.1186/s12859-017-1520-4 (PMC5324209; doi:10.1186/s12859-017-1520-4)
Supplement: Additional file 1: Table S1. — Summary of results and performance on bacteria other than Salmonella. (PDF 311 kb) [file 12859_2017_1520_MOESM1_ESM.pdf]

			Columns in Input			Maximum Compatible Set(s)		Ambiguities in Binary Columns		Columns Not Fully Disambiguated		Columns Represented on Tree			
Organism	Cluster number	Number of Genomes	Variable	Binary	Informative	Size	Number	Fraction Ambiguous States	Fraction of Columns with Ambiguities	Total	Informative and non-redundant	Number	Fraction of variable	Fraction of binary	Execution time (sec.)
Listeria	1	570	3447	3445	1274	3431	1	0.4%	20.6%	69	0	3362	97.5%	97.6%	0.56
	2	480	3043	3037	1190	3036	1	0.6%	6.2%	45	1	2991	98.3%	98.5%	0.47
	3	404	2440	2438	999	2431	1	0.1%	18.0%	17	0	2414	98.9%	99.0%	0.31
	4	300	2743	2739	1013	2735	1	0.1%	5.0%	13	0	2722	99.2%	99.4%	0.28
	5	280	889	889	451	887	1	0.1%	6.3%	20	9	867	97.5%	97.5%	0.17
	6	247	2284	2284	715	2278	1	0.1%	5.1%	6	1	2272	99.5%	99.5%	0.20
	7	245	839	839	240	835	1	0.5%	17.6%	44	0	791	94.3%	94.3%	0.15
	8	245	2131	2131	726	2130	1	1.1%	20.0%	81	2	2049	96.2%	96.2%	0.30
	9	192	1429	1429	648	1427	1	0.3%	15.3%	11	0	1416	99.1%	99.1%	0.26
	10	172	1511	1510	581	1499	1	0.3%	8.1%	17	1	1482	98.1%	98.1%	0.25
	11	164	1224	1224	414	1220	1	0.2%	7.4%	10	0	1210	98.9%	98.9%	0.26
	12	148	1176	1175	666	1171	1	0.3%	12.9%	4	0	1167	99.2%	99.3%	0.23
	13	147	1555	1553	482	1553	1	0.3%	10.9%	18	0	1535	98.7%	98.8%	0.26
	14	126	1120	1120	317	1116	1	0.7%	6.1%	18	0	1098	98.0%	98.0%	0.25
	15	125	217	217	49	217	1	1.4%	23.5%	26	3	191	88.0%	88.0%	0.18
	16	101	1051	1051	348	1051	1	0.1%	2.5%	4	1	1047	99.6%	99.6%	0.22
Escherichia coli	1	617	6005	6004	2537	5938	4	0.5%	29.8%	108.5	1.5	5830	97.1%	97.1%	1.17
	2	386	1891	1889	796	1731	4	1.2%	57.3%	54	5.5	1677	88.7%	88.8%	0.45
	3	372	3749	3746	1282	3710	1	1.7%	50.4%	178	5	3532	94.2%	94.3%	0.81
	4	307	3071	3067	1262	3030	2	2.1%	59.4%	287.5	1	2743	89.3%	89.4%	0.69
	5	250	2468	2468	854	2458	1	0.5%	17.7%	33	2	2425	98.3%	98.3%	0.33
	6	237	1074	1074	415	1066	1	0.6%	24.7%	31	5	1035	96.4%	96.4%	0.22
	7	230	984	984	356	977	1	0.5%	14.9%	20	1	957	97.3%	97.3%	0.23
	8	179	969	969	401	954	2	0.7%	25.6%	19	0	935	96.5%	96.5%	0.20
	9	143	96	96	9	96	1	1.4%	18.8%	17	3	79	82.3%	82.3%	0.42
	10	141	1044	1044	455	1031	1	2.0%	46.8%	123	0	908	87.0%	87.0%	0.28
	11	131	2062	2062	752	2043	1	0.6%	14.9%	17	0	2026	98.3%	98.3%	0.23
	12	116	69	69	39	69	1	0.1%	4.3%	1	0	68	98.6%	98.6%	0.17
	13	112	1303	1303	585	1290	1	0.7%	18.6%	20	1	1270	97.5%	97.5%	0.21
	14	108	690	689	286	687	1	0.4%	7.8%	10	0	677	98.1%	98.3%	0.23

	15	107	431	431	152	431	1	0.6%	19.3%	41	0	390	90.5%	90.5%	0.19
	16	102	808	808	389	804	1	0.9%	19.4%	25	0	779	96.4%	96.4%	0.19
	1	567	2768	2756	1101	2674	4	2.2%	88.4%	165.5	1.5	2509	90.6%	91.0%	0.88
Acinetobacter	2	154	396	395	164	395	1	0.3%	10.9%	3	0	392	99.0%	99.2%	0.21
	3	125	1326	1322	425	1287	1	0.8%	21.9%	19	0	1268	95.6%	95.9%	0.24
	1	195	818	818	274	798	1	2.9%	74.1%	36	3	762	93.2%	93.2%	0.20
Campylobacter	2	128	489	489	163	483	2	1.5%	57.3%	21	1	462	94.5%	94.5%	0.15
	3	103	662	661	175	648	1	3.3%	71.0%	74	5	574	86.7%	86.8%	0.16
Neisseria	1	259	1239	1237	579	1196	1	2.8%	65.5%	78	2	1118	90.2%	90.4%	0.26
	2	142	887	887	403	836	2	2.9%	58.1%	72.5	2.5	763.5	86.1%	86.1%	0.20
Vibrio parahaemolyticus	1	112	1516	1512	434	1473	2	0.8%	29.2%	23.5	0	1450	95.6%	95.9%	0.25
Klebsiella	1	296	2485	2478	889	2442	1	1.3%	34.3%	62	2	2380	95.8%	96.0%	0.49
Legionella pneumophila	1	108	586	586	152	581	1	0.8%	30.5%	26	0	555	94.7%	94.7%	0.21

Table S1. Summary of results and performance on bacteria other than *Salmonella* .

Some counts are averages over multiple maximum compatible sets, and may therefore be non-integral.
